# USP14 targets FABP5-mediated ferroptosis to promote proliferation and cisplatin resistance of HNSCC

**DOI:** 10.1007/s12094-025-03857-6

**Published:** 2025-02-10

**Authors:** Jiaxin Qian, Zitong Zhao, Liying Ma, Wensheng Liu, Yongmei Song

**Affiliations:** 1 State Key Laboratory of Molecular Oncology, National Cancer Center/National Clinical Research Center for Cancer/Cancer Hospital, Chinese Academy of Medical Sciences and Peking Union Medical College, Beijing, China; 2 Department of Head and Neck Surgical Oncology, National Cancer Center/National Clinical Research Center for Cancer/Cancer Hospital, Chinese Academy of Medical Sciences and Peking Union Medical College, Beijing, China

**Keywords:** Head and neck squamous cell carcinoma, Cisplatin resistance, USP14, FABP5, Ferroptosis

## Abstract

**Background:**

Head and neck squamous cell carcinoma (HNSCC) ranks among the most lethal solid tumors in humans, with a five-year survival rate hovering around 50%. The limited understanding of its biological foundation has hindered the development of efficacious targeted therapeutics.

**Methods:**

TCGA database and immunohistochemistry were deployed to confirm the expression levels of ubiquitin specific protease 14 (USP14). CCK8 method was used to evaluate the influence of USP14 on cisplatin resistance. Further investigations into the role of USP14 were conducted through assessments of cell proliferation, colony formation, and Transwell assays. The impact of USP14 expression on ferroptosis was evaluated by measuring GSH/GSSG ratios, Fe^2+^ concentrations, and lipid peroxide levels. Co-IP was employed to verify the interaction between USP14 and FABP5.

**Results:**

Our analysis revealed that USP14 ranked among the most prominently upregulated deubiquitinases (DUBs) in tissue samples of HNSCC. Notably, aberrant USP14 expression was linked to tumorigenesis and the malignant evolution of HNSCC and further suggested a poor prognosis. In vitro experiment revealed that USP14 depletion markedly inhibited cell growth, cisplatin resistance, invasion and migration capabilities of HNSCC cells. Mechanically, USP14 inhibits FABP5 ubiquitination and degradation, thus positively modulating FABP5 expression. Subsequent analyses demonstrated that the loss of USP14 promoted ferroptosis in HNSCC cells. Finally, in vivo xenograft experiments confirmed that the USP14 small molecular antagonist IU1 could effectively attenuate cisplatin resistance in HNSCC.

**Conclusion:**

The results indicate that the USP14-FABP5 axis exerts oncogenic effects on HNSCC, providing a potential target for diagnosing and treating this type of malignancy.

**Supplementary Information:**

The online version contains supplementary material available at 10.1007/s12094-025-03857-6.

## Introduction

HNSCC is the sixth most prevalent malignant tumors globally, characterized by a high incidence and mortality rate. The approximate 5-year survival rate of HNSCC is 50% [1, 2]. Major risk factors for HNSCC are smoking, drinking alcohol, and contracting HPV [3, 4]. Currently, the principal therapeutic approaches are surgery combined with radiotherapy, chemotherapy, molecular targeted therapies, or immunotherapy [5–7]. Cisplatin (CDDP)-based chemotherapy has been established as the classic treatment option for HNSCC [8]. Nevertheless, a subset of patients exhibit resistance to cisplatin, which greatly limits its efficacy and adversely impacts prognosis [9]. Therefore, studies elucidating the molecular mechanisms of chemoresistance and developing novel therapeutic strategies are urgently needed.

Cisplatin is a foundational first-line chemotherapy drug for HNSCC, and it exerts its effects by blocking DNA replication in cancer cells. Several viewpoints on the mechanisms of cisplatin resistance have been previously proposed, including increased repair of DNA damage [10], suppression of apoptosis, upregulation of stemness signalling [11], and activation of cellular antioxidative defenses [12]. Studies have shown abnormal strengthening of the antioxidant defense system linked to chemotherapy resistance in numerous cancer types in the past few years. Cisplatin can induce cells to generate reactive oxygen species (ROS), which triggers cell death. However, cancer cells can upregulate GPX4, NRF2 and other pathways to clear intracellular ROS, thus inhibiting ferroptosis of tumor cells [13, 14]. In addition, it was found that targeting antioxidant pathways, including SGK3-catalase axis [15] and growth differentiation factor 15 (GDF15) [16], could restore cisplatin sensitivity depending on the manner of oxidative stress. Presently, the activation of ferroptosis emerges as a promising therapeutic target to subvert clinical resistance across a broad spectrum of cancers [17, 18].

The ubiquitin–proteasome system (UPS) including DUBs regulates approximately 90% of proteins’ stability. DUBs are considered important regulators of oncogenes and tumor suppressor genes because they inhibit protein degradation by detaching the ubiquitin tag from the substrate protein [19]. The human genome encodes about 100 DUBs, which can be divided into five families: Ubiquitin Specific Proteases (USPs), Ubiquitin C-Terminal Hydrolases (UCHs), Ovarian Tumor Proteases (OTUs), Machado-Josephin Domain Proteins (MJDs), and JAB1/MPN/MOV34 Metalloenzymes (JAMMs). DUBs play important regulatory roles in cellular processes such as cell growth, proliferation, apoptosis, DNA repair, modulation of kinase activity, and regulation of transcription [20–22]. USP14 is a member of the USPs subfamily and is functionally related to the 26S proteasome. The USP14 protein is 494 amino acids long and is divided into two primary domains: ubiquitin like domain (UBL) and catalytic domain (CAT) [23, 24]. Contemporary research has revealed the overexpression of USP14 in many types of cancer [25]. Its abnormal activity is strongly connected to the onset, progression and drug resistance of cancer [26]. However, the mechanism by which USP14 affects tumorigenesis and development remains unclear and requires additional study.

In this study, we elucidated that USP14 was associated with a malignant phenotype and cisplatin resistance in HNSCC cells. Our research demonstrated that USP14 augmented the proliferation as well as metastatic dissemination of HNSCC cells while repressing ferroptosis. USP14 stabilizes FABP5 protein through the mechanism of deubiquitination. The findings indicate that the USP14–FABP5 axis supports the malignant characteristics observed in HNSCC cells.

## Methods

### TCGA data analysis and tissue microarray IHC staining

The transcript levels of USP14 were retrieved from TCGA database for both HNSCC tissues and normal tissues. Tissue microarrays (HN117Oc01) containing HNSCC tissues (*n* = 88) and normal head and neck tissues (*n* = 10) were obtained from Bioaitech (Xi’an, China). Immunohistochemical detection of USP14 was performed according to standard procedures. USP14 antibody (#14,517–1-AP, Proteintech, Wuhan, China) was diluted at 1:200. We divided the samples into four categories according to percentage of cells stained positively: 0 to 1 (0–25%), 1 to 2 (26–50%), 2 to 3 (51–75%), and 3 to 4 (76–100%). To access staining intensity, we categorized it into 0–3 four distinct levels, which were no appreciable staining; weak intensity (light yellow); moderate intensity (yellow‒brown) and strong intensity (brown). Then we calculated the staining index by multiplying the score for the number of cells with positive staining by the score for the intensity of staining. The samples were categorized into two groups exhibiting either high or low expression levels based on the average protein expression scores.

### Cell lines and cell culture

The cell lines utilized in the research were obtained from the State Key Laboratory and were preserved in liquid nitrogen. The cells were cultured in an atmosphere of 95% air and 5% CO2 in DMEM or RPMI 1640 (Gibco). The medium was previously supplemented with 1% P/S (Penicillin–Streptomycin Solution) and 10% FBS (fetal bovine serum). Besides, we sent cell lines to the Biotech Company for test, with results confirmed their negative status of mycoplasma contamination.

### Cell transfection and lentiviral transfection

The construction of small interfering RNAs (siRNAs) and plasmids was facilitated by generay (Shanghai, China), GenePharma (Shanghai, China) and Ribobio (Guangzhou, China). [siUSP14_1: (sense) GCAAAGAAAUGCCUUGUAU, (antisense) AUACAAGGCAUUUCUUUGC; siUSP14_2: (sense) GGCUCCAAUAAUUGUGGAU, (antisense) AUCCACAAUUAUUGGAGCC]. siRNA transfection was performed using Lipofectamine 2000 (Thermo Fisher Scientific). Neofect™ DNA transfection reagent (NEOFECT, Beijing, China) was utilized for plasmid transfection following the provided protocols. Furthermore, lentiviral vectors for USP14 overexpression along with a control were obtained from GeneChem (China). HNSCC cells transfected for 48 h, and western blotting and qRT-PCR verification were performed.

### Western blotting

These assays were conducted as previously described [27] using the following antibodies: anti-USP14, anti-FABP5, anti-GPX4, anti-SLC7A11 (Proteintech, China), anti-Ubiquitin (Santa Cruz Biotechnology, USA), anti-β-actin and anti-GAPDH (Cell Signaling Technology, USA).

### RNA extraction and real-time quantitative PCR (qPCR)

These assays were conducted as previously described [27]. Normalization of data was achieved by β-actin, and the outcomes were depicted as relative mRNA levels. The sequences of primers used were as follows: USP14-F: GCTGTTTGCGTTGACTGGAG; USP14-R: GCCATTTCCCCTGAAGCTCT; FABP5-F: AGGAGTGGGAATAGCTTTGCG; FABP5-R: GCTGAAC CAATGCACCATCT.

### Coimmunoprecipitation (Co-IP)

In order to identify protein interactions with USP14, CAL27 cells underwent lysis using IP lysis buffer and were completely lysed through a vortex. Subsequently, the protein lysate was centrifugated at 120,000 r for 20 min to purify. The supernatants containing 2 mg proteins were incubated with either anti-USP14 or IgG (Proteintech, SA00001-2) on a shaker at 4 °C overnight. Then, we added 80 μL protein A/G beads (Santa Cruz, sc-2003), followed by a 4-h incubation. After that, the mixtures of reactions were centrifuged at 3000r for 5 min, following with a minimum of three washes using washing buffer. The peptide fragments interacting with USP14 were identified using a mass spectrometer. We then used Co-IP followed by immunoblotting to determine protein interactions.

### Cell proliferation and colony forming assays

After resuspending the cells, they were counted using the TC10 Automated Cell Counter (Bio-Rad, California, USA). We first added 100 μL of medium to the 96 E-Plate for baseline detection, and then added 2000 cells/100 μL to each well. The xCELLigence Real-Time Cell Analyzer-MP system (ACEA, Agilent Technologies, California, USA) was utilized to measure the cell index of HNSCC cells. For the colony-forming assay, we seeded HNSCC cells in 6-well plates at a density of 1000 cells per well for incubation. Colonies would form over a period of approximately 2 weeks. Then, we stained the colonies with crystal violet (Beyotime, C0121), followed by quantification of the colony numbers in each group using image J.

### Transwell assay

The experiment on cell migration was carried out in the following manner. A Transwell system (Corning, New York, USA) was utilized, where 1 × 10^5 cells were seeded in the upper chamber containing 200 μL FBS-free medium. 700 μL DMEM with 20% FBS was added to the booton chamber. After approximately 8 h to 24 h of incubation, the upper chambers were stained with 2% crystal violet. We removed cells on the top surface with a cotton swab. Next, the cells on the underside were enumerated with a microscope. The cell invasion assay was performed similarly, using a transwell membrane that had been precoated with 100 μL 2% Matrigel.

### Cell viability assay

The viability of the cells was evaluated utilizing the Cell Counting Kit-8 (CCK-8, APExBIO, K1018) assay. To begin, 10% CCK-8 solution in DMEM complete medium was applied to a 96-well plate. The spectrometer was used to measure the optical density (OD) at 450 nm after incubation for 3 h at 37 °C. Each well was assessed in three independent readings to ensure accuracy.

### ROS and antioxidant capacity detection

HNSCC cells were pretreated with Rosup reagent or cisplatin. Subsequently, the cells were incubated with DCFH-DA (Beyotime, S0033S) and washed three times with PBS. The detection of DCF fluorescence signals was conducted using flow cytometry. The obtained FITC signals were subsequently analyzed and visualized with Flow Jo software version 10.6.2. The ratio of glutathione to oxidized glutathione (GSH/GSSG) was determined through the application of GSH/GSSG detection assay (Beyotime, S0053) and a Microplate Reader according to the protocols. Besides, we incubated the cells with ferroOrange (Dojindo, F374) or Liperfluo (Dojindo, L248) reagent according to the protocols. After that, Fe^2+^ level or lipid peroxide levels was evaluated with confocal microscope and image J.

### Animal experiments

Beijing Vital River Laboratory provided mice that were kept in standard specific-pathogen-free (SPF) conditions. We randomly divided twenty-four 5-week-old male BALB/c nude mice into four groups with 6 mice in each group. The appropriate CAL27 cells (approximately 2 × 10^6^) were injected subcutaneously into the right flanks of the mice to establish the xenograft model. Subsequently, the mice received IU1 (MedChemExpress, HY-13817) at a dosage of 40 mg/kg in 150μL of corn oil via intragastric administration and cisplatin (1 mg/kg) by intraperitoneal injection every 3 days. The volume of tumor was systematically monitored at 3-day intervals. The measurements were then used to calculate the tumor size with a common equation: 0.5 × Length × Width^2. Following six times of injection, the tumors were subsequently gathered to be weighed and examined using immunohistochemistry (IHC).

### Statistical analysis

Continuous data were displayed as mean ± SD and assessed using Student’s *t* test for comparisons between two groups and one-way ANOVA for comparisons among multiple groups. GraphPad Prism 8.0 was utilized to analyze data and draw statistical graphs. Image J was used for image assessment. *P* < 0.05 indicated statistical significance.

## Results

### The expression level of USP14 is high in HNSCC tissues and is relevant to cisplatin resistance

**Fig. 1:**
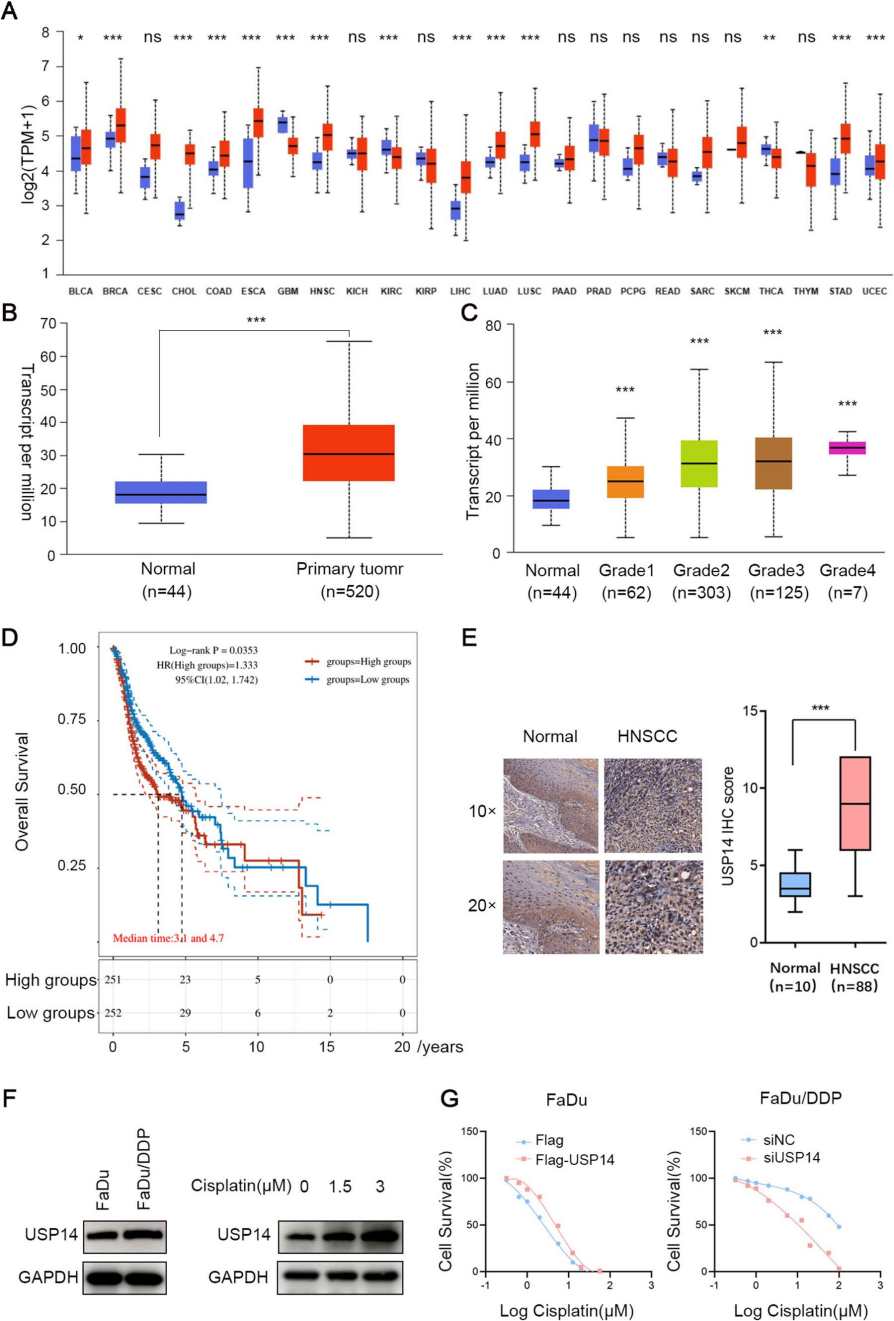
The expression level of USP14 is high in HNSCC tissues and is relevant to cisplatin resistance. **A** The expression levels of the USP14 mRNA in different tumors and normal tissues from TCGA database. **B** USP14 mRNA expression in HNSCC tissues and normal tissues from TCGA database. **C** USP14 mRNA expression in HNSCC tissues according to different grades. **D** Comparison of probability of patients’ survival with high or low USP14 expression level using Log-rank test. **E** Representative immunohistochemistry (IHC) images showing the distribution of USP14 in HNSCC or normal head and neck tissues. **F** Western blot results showing USP14 expression levels in FaDu and FaDu/DDP cells, as well as USP14 expression levels after treatment with different concentrations of cisplatin. **G** Cisplatin IC50 evaluations in USP14-overexpressing FaDu cell or USP14-knockdown FaDu/DDP cell. Data are presented as the mean ± SD. *ns* not significant, *P < 0.05; **P < 0.01; ***P < 0.001

In order to investigate the impact of deubiquitinating proteins related to ferroptosis on the progression of HNSCC, we analyzed the expression of 25 ferroptosis-related genes in HNSCC using the TCGA database and found that 14 of these genes are highly expressed in HNSCC. Next, we conducted a correlation analysis between these highly expressed genes and the USP gene families. Most of ferroptosis-related genes were in positive correlation with USP14 (Supplementary Fig. 1). We first used TCGA datasets to detect USP14 mRNA expression in 24 solid tumors and corresponding normal controls. Among these cancers, 14 demonstrated significant dysregulation of USP14 transcription level. In addition, analysis of the TCGA-HNSCC cohort dataset revealed that USP14 was markedly upregulated in HNSCC tissue samples in comparison with noncancerous tissue samples (Fig. 1B). Notably, we observed a strong correlation between USP14 mRNA levels and tumor grade; low USP14 expression prevalent in grade 1/2 tumors, while high USP14 expression was characteristic of grade 3/4 tumors (Fig. 1C). Moreover, to assess the prognostic significance of USP14 in HNSCC, we investigated its association with overall survival (OS). Kaplan–Meier analysis demonstrated that elevated USP14 expression was linked to worse OS in HNSCC patients (Fig. 1D). Immunohistochemical (IHC) staining revealed increased USP14 in HNSCC tissues (n = 88) compared with noncancerous tissue samples (*n* = 10) (Fig. 1E). Moreover, western blot analysis revealed higher USP14 expression in FaDu/DDP cell than that in FaDu cell, and USP14 was elevated in HNSCC cells in a cisplatin-concentration-dependent manner (Fig. 1F). And the IC50 results confirmed that elevated levels of USP14 led to enhanced resistance to cisplatin in FaDu cells, while reducing USP14 levels decreased resistance to cisplatin in FaDu/DDP cells (Fig. 1G).

### USP14 enhances the proliferation, colony formation, migration and invasion of HNSCC cells

**Fig. 2:**
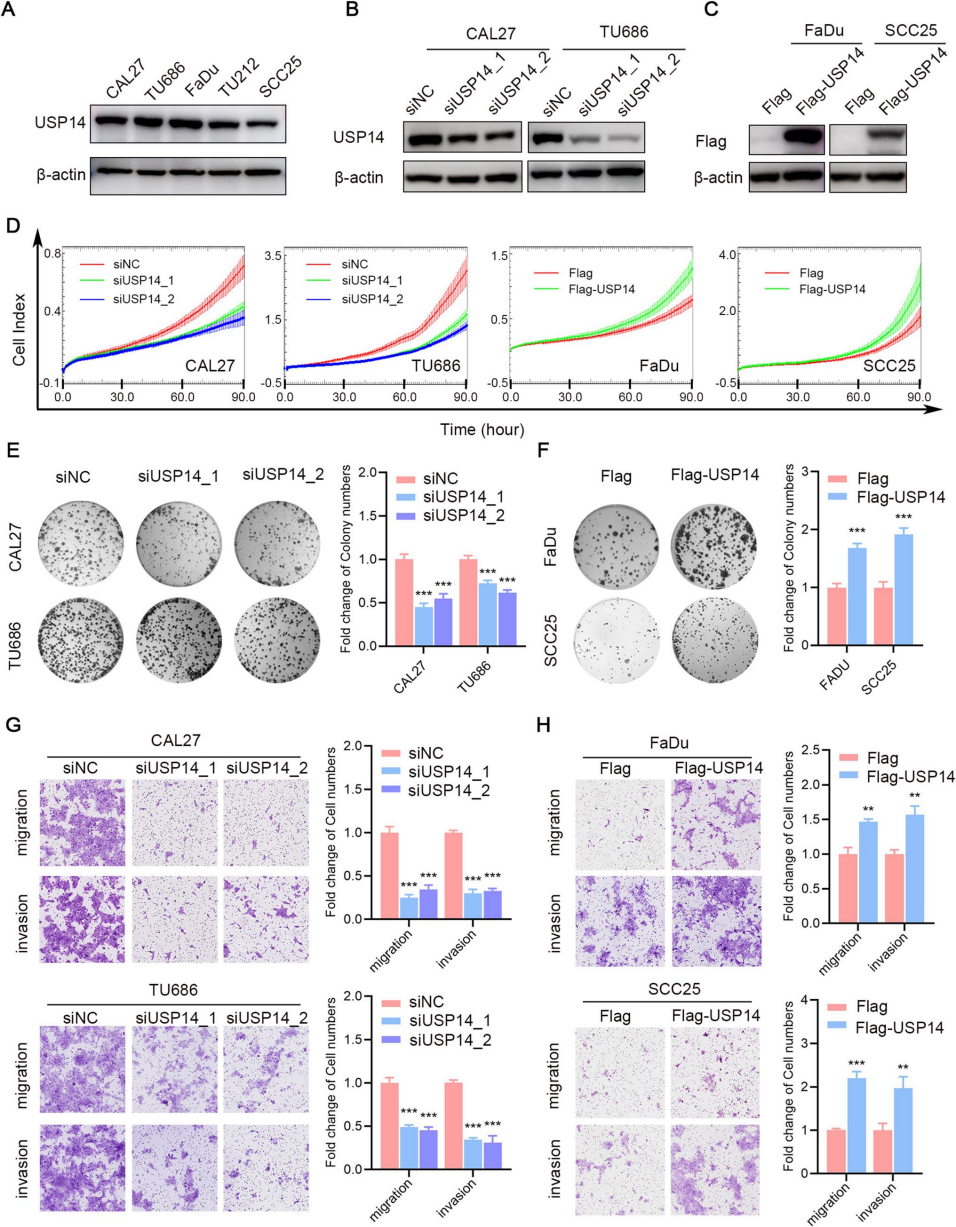
USP14 enhances the proliferation, colony, migration and invasion of HNSCC cells. **A** The expression of USP14 in five HNSCC cell lines detected by western blot. **B** Western blot results showing USP14 expression levels in HNSCC cell lines (CAL27 and TU686) transfected with either NC or USP14-specific siRNA. **C** Western blot results showing USP14 expression levels in HNSCC cell lines (FaDu and SCC25) transfected with either Vector-Flag or Flag-USP14 plasmids. **D** Cell proliferation assay results of HNSCC cells with USP14 overexpression or knockdown. **E**, **F** Colony formation assay of HNSCC cell lines after knockdown **E** or overexpression **F** of USP14. The number of colonies is presented in bar plots. **G**, **H** Transwell assays were used to test the migration and invasion ability of HNSCC cell lines, and ImageJ software was used to quantify the number of cells. Data are presented as the mean ± SD. *ns* not significant; *P < 0.05; **P < 0.01; ***P < 0.001

To assess whether USP14 controls HNSCC cell growth, we analyzed USP14 protein levels in five HNSCC cell lines, namely, CAL27, TU686, FaDu, TU212 and SCC25 cells (Fig. 2A). Additionally, we assessed USP14 knockdown and overexpression efficacy through western blot analysis (Fig. 2B, C). Subsequently, we explored the association between USP14 expression and HNSCC cell proliferation through the following steps. Real Time Cellular Analysis (RTCA) revealed USP14 silencing reduced the growth of HNSCC cells. Consistent with the results of USP14 knockdown, USP14 overexpression increased the viability of HNSCC cells (Fig. 2D). Colony formation assays revealed that USP14 depletion suppressed colony formation and that USP14 upregulation enhanced colony formation (Fig. 2E, F), collectively suggesting USP14 plays a pivotal regulatory role in HNSCC cell proliferation. Additionally, to elucidate the involvement of USP14 in HNSCC cell migration and invasion, Transwell assays were conducted. The findings indicated that the level of USP14 expression impacted the motility of HNSCC cells, with USP14-depleted cells exhibiting significantly reduced migration and invasion compared to control cells (Fig. 2G). Conversely, USP14 overexpression notably heightened migration and invasion abilities in FaDu and SCC25 cells (Fig. 2H). These data indicated USP14 could enhance the metastasis of HNSCC.

### USP14 binds to FABP5 and induces FABP5 deubiquitination

**Fig. 3:**
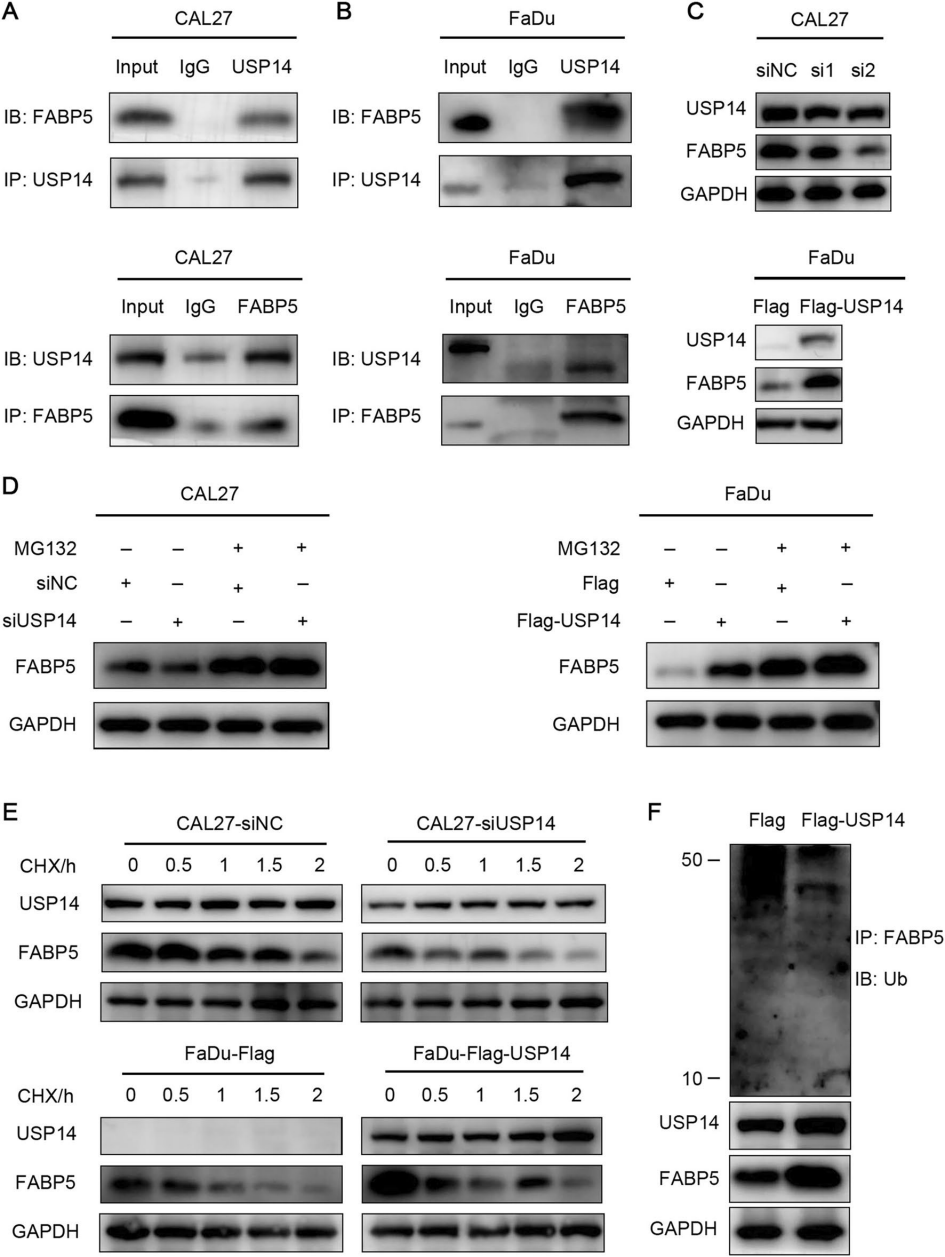
USP14 binds to FABP5 and induces FABP5 deubiquitination. **A**, **B** Co-IP assay of the interaction of USP14 and FABP5 in HNSCC cell lines. **C** Western blot showing the FABP5 level after USP14 depleting or overexpressing in HNSCC cell lines. **D** Protein expression levels of FABP5 after MG132 (10 μM) treatment for 12 h in USP14 siRNA-treated CAL27 cells or USP14-overexpressing FaDu cells by Western blotting. **E** Protein expression levels of FABP5 after CHX (10 μg/mL) treated for the indicated time points with USP14 knockdown or overexpression by Western blotting. **F** The USP14-overexpressing cells and control cells were treated with MG132 (10 μM) for 12 h. The polyubiquitinated (PolyUb) FABP5 protein was analyzed by western blot with anti-ubiquitin after Co-IP

Subsequently, we elucidated the potential molecular mechanism by which USP14 suppressed the proliferation and spread of HNSCC cells. To identify downstream targets of USP14, immunoprecipitation was performed in conjunction with mass spectrometry analysis. We aimed to study the protein related to ferroptosis. FABP5 is a fatty acid-binding protein, and we speculated that it may be related to ferroptosis. Additionally, it is reported recently that FABP5 can inhibit ferroptosis in cancers [28, 29]. Supplementary Table 1 showed some of the proteins with high scores in the mass spectrometry results. Besides, we made a table of FABP5 expression and clinical patterns according to the result of immunohistochemistry. FABP5 was associated with some adverse prognostic factors in HNSCC such as T stage and lymph node metastasis (Supplementary Table 2). The outcomes of the mass spectrometry experiments suggested a potential interaction between USP14 and FABP5. To confirm the actual relationship between USP14 and FABP5, coimmunoprecipitation assays were conducted to assess the binding affinity of these proteins. In cell lysates, a substantial quantity of FABP5 was coprecipitated with USP14. Conversely, USP14 was also observed to coprecipitate with FABP5, indicating a significant interaction between these two proteins (Fig. 3A, B). We then assessed whether and how USP14 regulates FABP5 within various cellular contexts. The expression of FABP5 protein was significantly elevated in CAL27 and FaDu cells with USP14 overexpression. Conversely, the FABP5 protein level was significantly decreased in cells with USP14 knockdown (Fig. 3C). These findings suggested that USP14 positively modulates the protein level of FABP5. Given the known function of USP14 as a deubiquitinating enzyme, it was hypothesized that USP14 mediates the deubiquitination of FABP5, thereby impeding its degradation. USP14 knockdown led to a substantial decrease in USP14 protein levels, which were then replenished in cells exposed to the proteasome inhibitor MG132 (Fig. 3D). To further explore the influence of USP14 on FABP5 stability, HNSCC cells were exposed to cycloheximide (CHX), a protein synthesis inhibitor. USP14 depletion hastened the breakdown of FABP5 protein in CAL27 cells, while FABP5 degradation was suppressed in cells overexpressing USP14 (Fig. 3E). Finally, our experiments revealed that USP14 overexpression in CAL27 cells resulted in reduced ubiquitination levels of FABP5, whereas vector-transduced control cells exhibited elevated levels of ubiquitinated FABP5, confirming the role of USP14 in promoting FABP5 deubiquitination (Fig. 3F).

### USP14 positively regulates the expression of FABP5

**Fig. 4:**
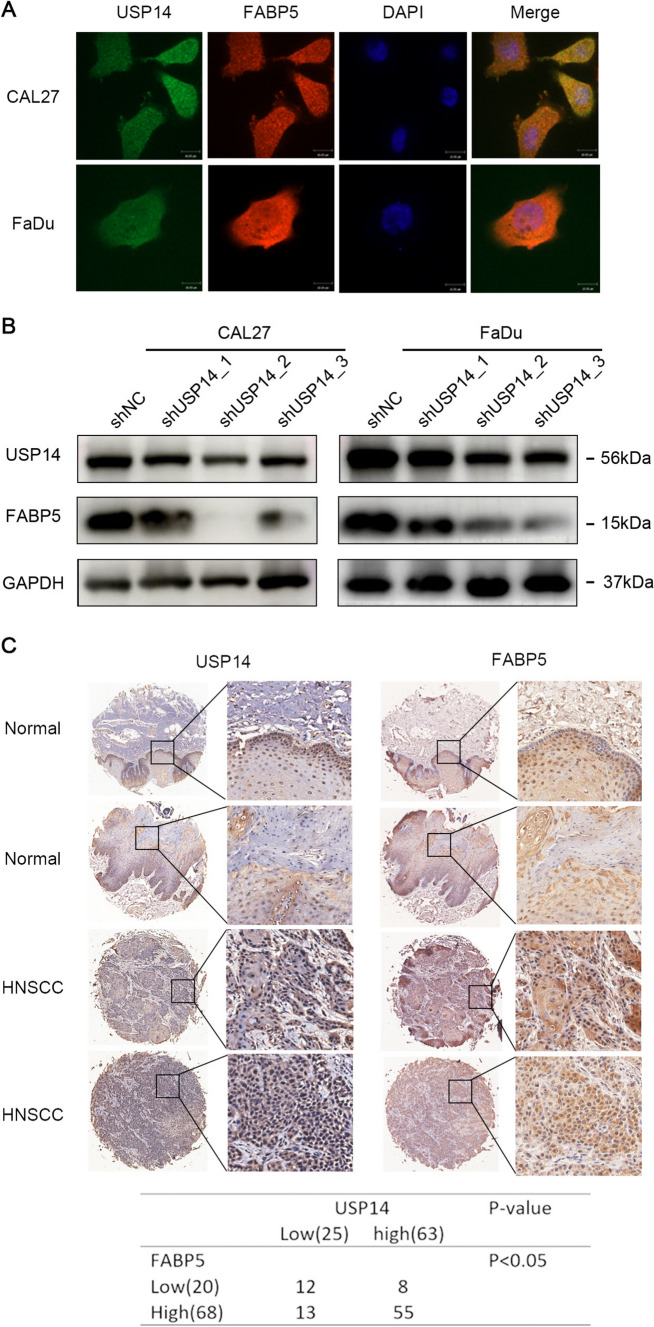
USP14 positively regulates the expression of FABP5. **A** Colocalization of USP14 and FABP5 in CAL27 and FaDu by immunofluorescence. Scale bar, 100 μm. **B** Western blotting showing the FABP5 level after USP14 knockdown. **C** Representative IHC images of USP14 in the normal head and neck tissue array and HNSCC tissue array

To confirm the correlations of USP14 and FABP5 in HNSCC, an initial immunofluorescence analysis was conducted. This analysis aimed to determine the cellular localization of both USP14 and FABP5, providing crucial insight into their spatial distribution within the cells (Fig. 4A). This examination revealed the colocalization of USP14 and FABP5 within the cells. Subsequently, to evaluate the expression levels of FABP5 in cells with stable knockdown of USP14, western blotting was employed. This analysis revealed a significant reduction in FABP5 expression following the decreased expression of USP14, thereby suggesting a direct regulatory influence of USP14 on FABP5 levels (Fig. 4B). Immunohistochemical evaluations further demonstrated that FABP5 expression was elevated in HNSCC specimens relative to normal tissue specimens, and a positive correlation was identified between the expressions of USP14 and FABP5 in HNSCC tissues (Fig. 4C).

### USP14 inhibits ferroptosis of HNSCC cells

**Fig. 5:**
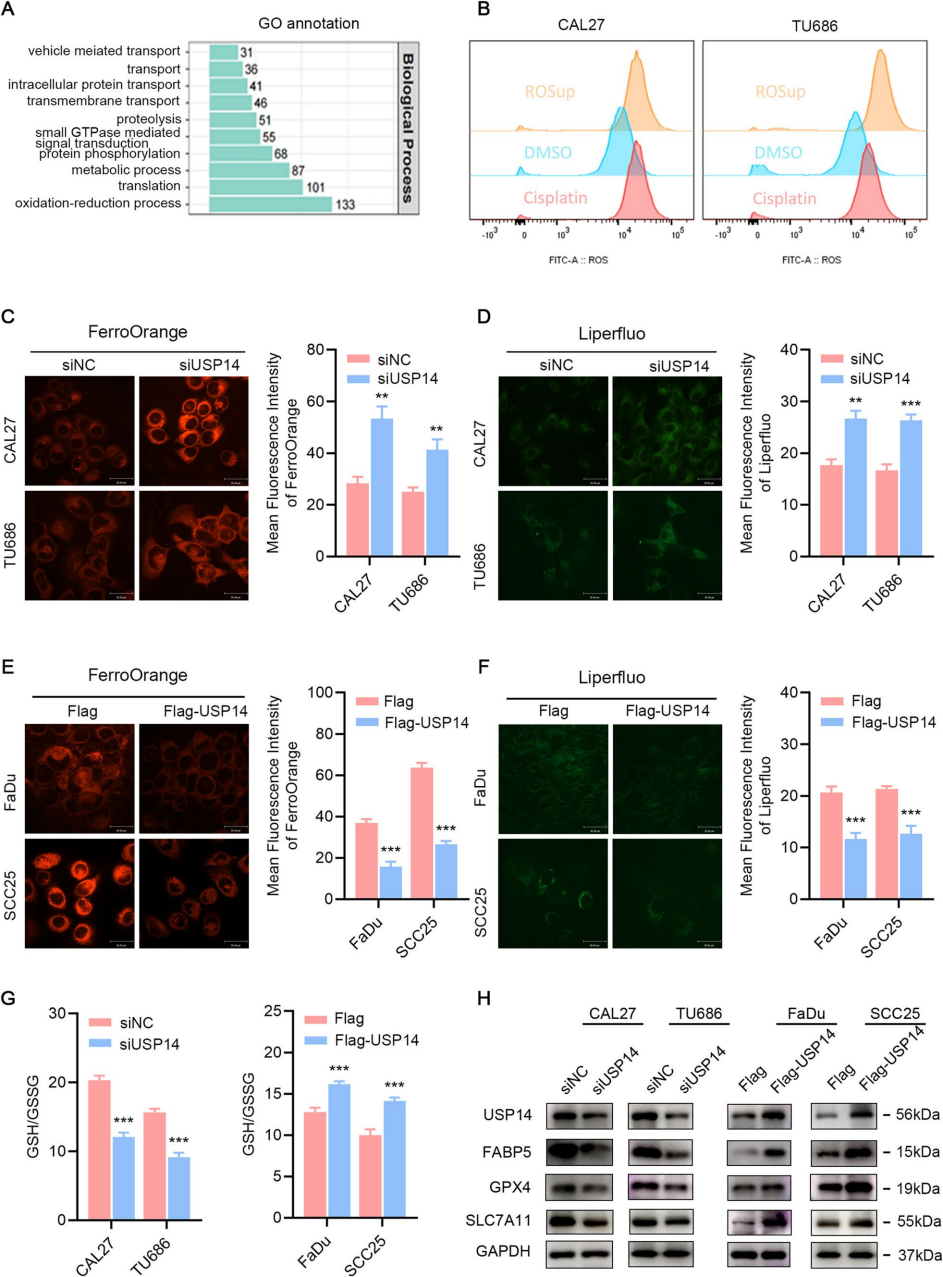
USP14 inhibits ferroptosis of HNSCC cells. **A** Top 10 biological process terms enriched in GO analysis. **B** Flow cytometry analyses of ROS in ROSup, DMSO or cisplatin treatment in CAL27 and TU686 cells. **C**, **E** Fe^2+^ levels in HNSCC cells with USP14 knockdown or overexpressing detected by FerroOrange assay and confocal microscope. **D**, **F** Lipid peroxides levels in HNSCC cells with USP14 knockdown or overexpressing detected by Liperfluo assay and confocal microscope. **G** GSH/GSSG ratio in HNSCC cells with USP14 knockdown or overexpressing. **H** Western blot of indicated proteins in the USP14 overexpressing or knockdown cells and corresponding control cells. Data are presented as the mean ± SD. *ns* not significant, *P < 0.05; **P < 0.01; ***P < 0.001

To further elucidate the biological role of USP14, Gene Ontology (GO) analysis was conducted, revealing a significant association with the oxidation–reduction process (Fig. 5A). Flow cytometric assessment showed higher levels of reactive oxygen species (ROS) after cisplatin treatment in HNSCC cells (Fig. 5B). Given that USP14 has been linked to cisplatin resistance in HNSCC as mentioned above, we hypothesized that USP14 may promote tumor progression by reducing ferroptosis through ROS clearance.

The experimental downregulation of USP14 increased the levels of ferrous ions (Fe2 +) and lipid peroxides (Fig. 5C, E), concurrently decreased the GSH/GSSG ratio (Fig. 5G). In contrast, USP14 overexpression decreased Fe^2+^ and lipid peroxides (Fig. 5D, F) and increased the GSH/GSSG ratio (Fig. 5G). The protein levels of FABP5, SCL7A11 and GPX4 were significantly reduced after USP14 knockdown, while the levels of these proteins were increased following USP14 overexpression assessed with western blotting (Fig. 5H). Cumulatively, these results suggest a pivotal function of USP14 in alleviating ferroptosis within HNSCC cells.

### FABP5 overexpression reversed the functional effects of USP14 knockdown

**Fig. 6:**
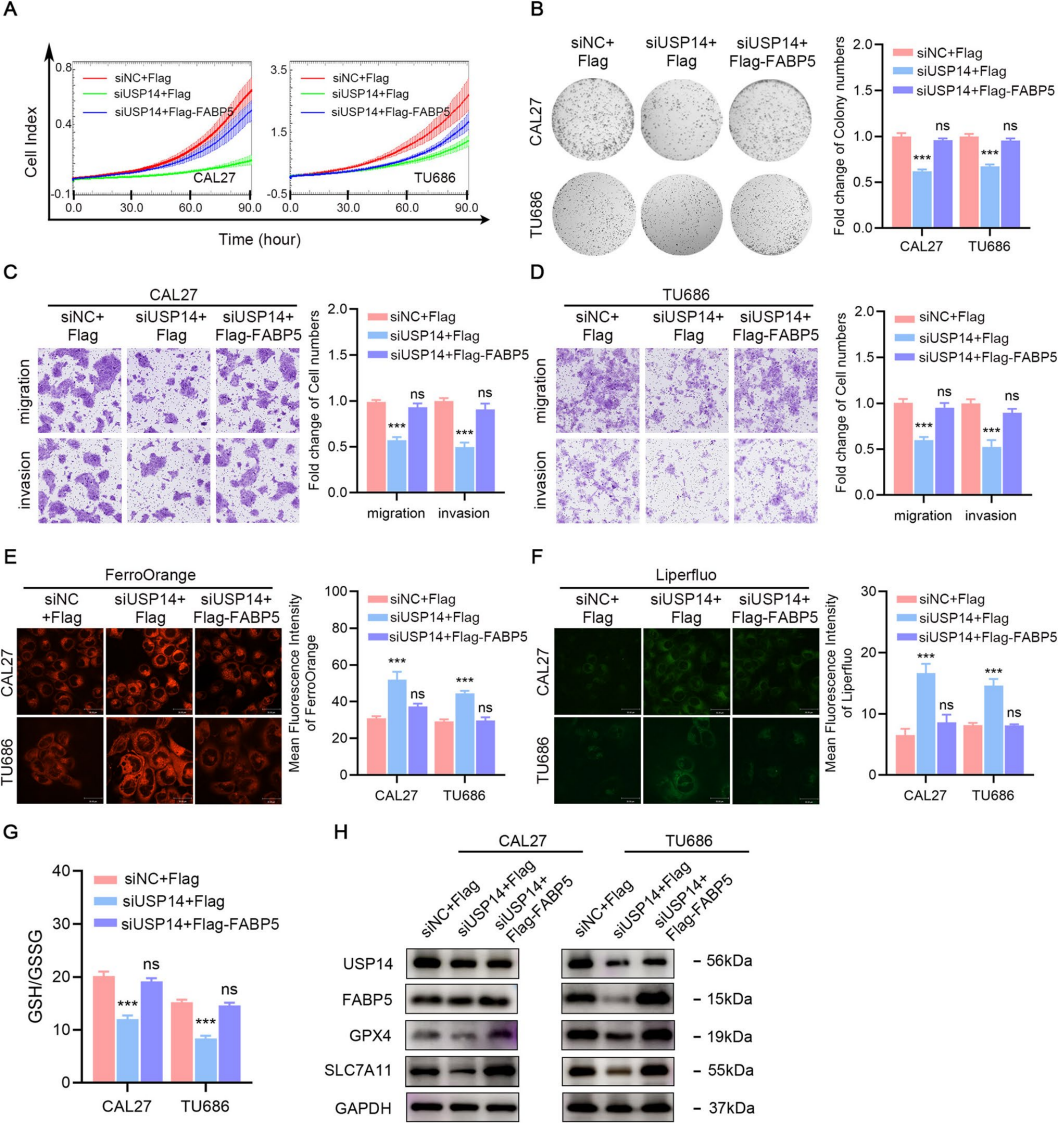
FABP5 overexpression reversed the functional effects of USP14 knockdown. **A** Cell proliferation of USP14 knockdown HNSCC cells with or without FABP5 overexpressing. **B** Colony formation of USP14 knockdown HNSCC cells with or without FABP5 overexpressing. **C**, **D** Migration and invasion ability of USP14 knockdown HNSCC cells with or without FABP5 overexpressing. **E** Fe^2+^ levels in USP14 knockdown HNSCC cells with or without FABP5 overexpressing. **F** Lipid peroxides levels USP14 knockdown HNSCC cells with or without FABP5 overexpressing. **G** GSH/GSSG ratio of USP14 knockdown HNSCC cells with or without FABP5 overexpressing. **H** Western blot of indicated proteins. Data are presented as the mean ± SD. *ns* not significant; *P < 0.05; **P < 0.01; ***P < 0.001

Although the preceding findings identified FABP5 as a downstream target of USP14 for deubiquitination, it remains unclear whether this is a regulatory mechanism by which USP14 promotes proliferation and inhibits ferroptosis in HNSCC cells. FABP5 has been reported to inhibit ferroptosis in cancer cells [28, 29]. Furthermore, our previous findings showed that suppressing USP14 can impede the proliferation and metastasis of HNSCC cells while also enhancing ferroptosis. Interestingly, when we knocked down USP14 and overexpressed FABP5 at the same time, it was found that overexpression of FABP5 could restore the inhibitory effect of USP14 on various cellular processes, including proliferation, colony, migration and invasion of HNSCC cells (Fig. 6A–D). Furthermore, knockdown of USP14 promoted ferroptosis as reflected by Fe2^+^ levels, lipid peroxides levels and GSH/GSSG ratios, while FABP5 overexpression restored these ferroptosis indicators to control levels (Fig. 6E–G). We assessed the expression of USP14, FABP5, GPX4 and SLC7A11 by immunoblotting in HNSCC cells. Downregulation of USP14 by siRNA suppressed the protein expression, which was reversed in CAL27 or TU686 cells cotransfected with USP14 siRNA and FABP5 overexpression plasmid (Fig. 6H).

In summary, these results collectively confirmed that FABP5 overexpression effectively reversed the functional effects associated with USP14 knockdown, substantiating the role of FABP5 as a downstream target of USP14 in regulating HNSCC cell proliferation, metastasis and ferroptosis.

### Effects of IU1 and cisplatin on tumor growth in vivo

**Fig. 7:**
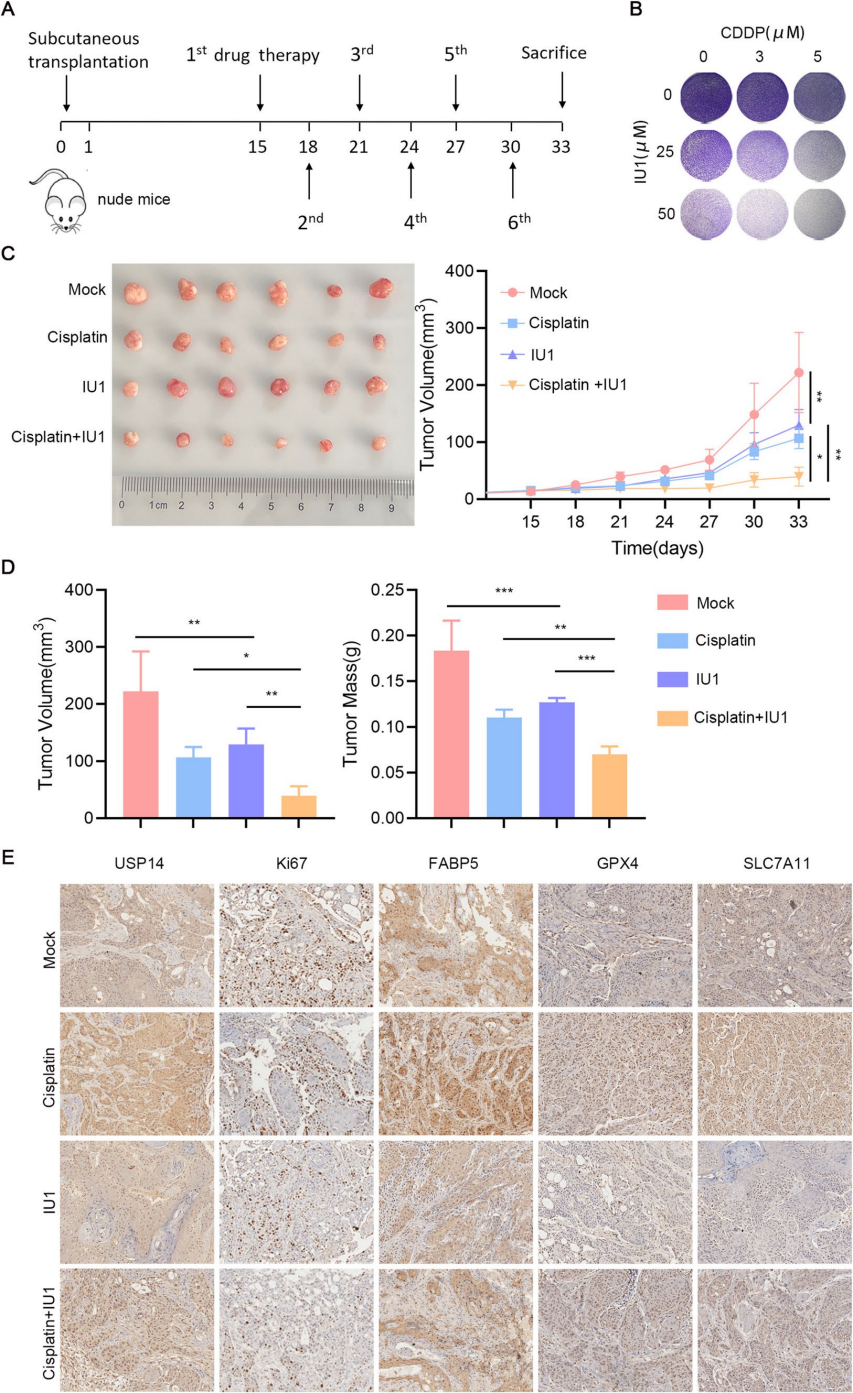
Effects of IU1 and cisplatin on tumor growth in CAL27 xenograft model. **A** Schematic of mouse studies. **B** Combined efficacy of cisplatin and IU1 in CAL27 cell. **C** Representative tumor images of each group (*n* = 6) at the end of treatment. Curves of change in tumor volume for each group. **D** Bar chart of tumor volume and tumor mass at the end of the experiment. **E** Representative immunohistochemistry images of USP14, ki67, FABP5, GPX4, SLC7A11 in CAL27 xenografts. Data are presented as the mean ± SD. *ns* not significant; *P < 0.05; **P < 0.01; ***P < 0.001

A subcutaneous xenograft model employing CAL27 cells was established for further experiments to elucidate the therapeutic potential of IU1 (a small molecule inhibitor of USP14) in reversing cisplatin resistance. The tumor volume, growth rate and weight in the IU1 or cisplatin treatment groups were lower. Notably, the concomitant administration of IU1 and cisplatin exhibited the most pronounced therapeutic efficacy on the tumors, indicating that IU1 could increase the sensitivity of HNSCC tumors to cisplatin therapy (Fig. 7A–D). Besides, by contrast with those in the cisplatin treatment group, immunohistochemical analysis showed that the expressions of USP14, FABP5, proliferation factor (Ki-67) and ferroptosis-related molecules (GPX4 and SLC7A11) were decreased after combination treatment of cisplatin and IU1 (Fig. 7E). In conclusion, these findings indicate that IU1 increased cisplatin sensitivity in HNSCC xenografts.

## Discussion

**Fig. 8:**
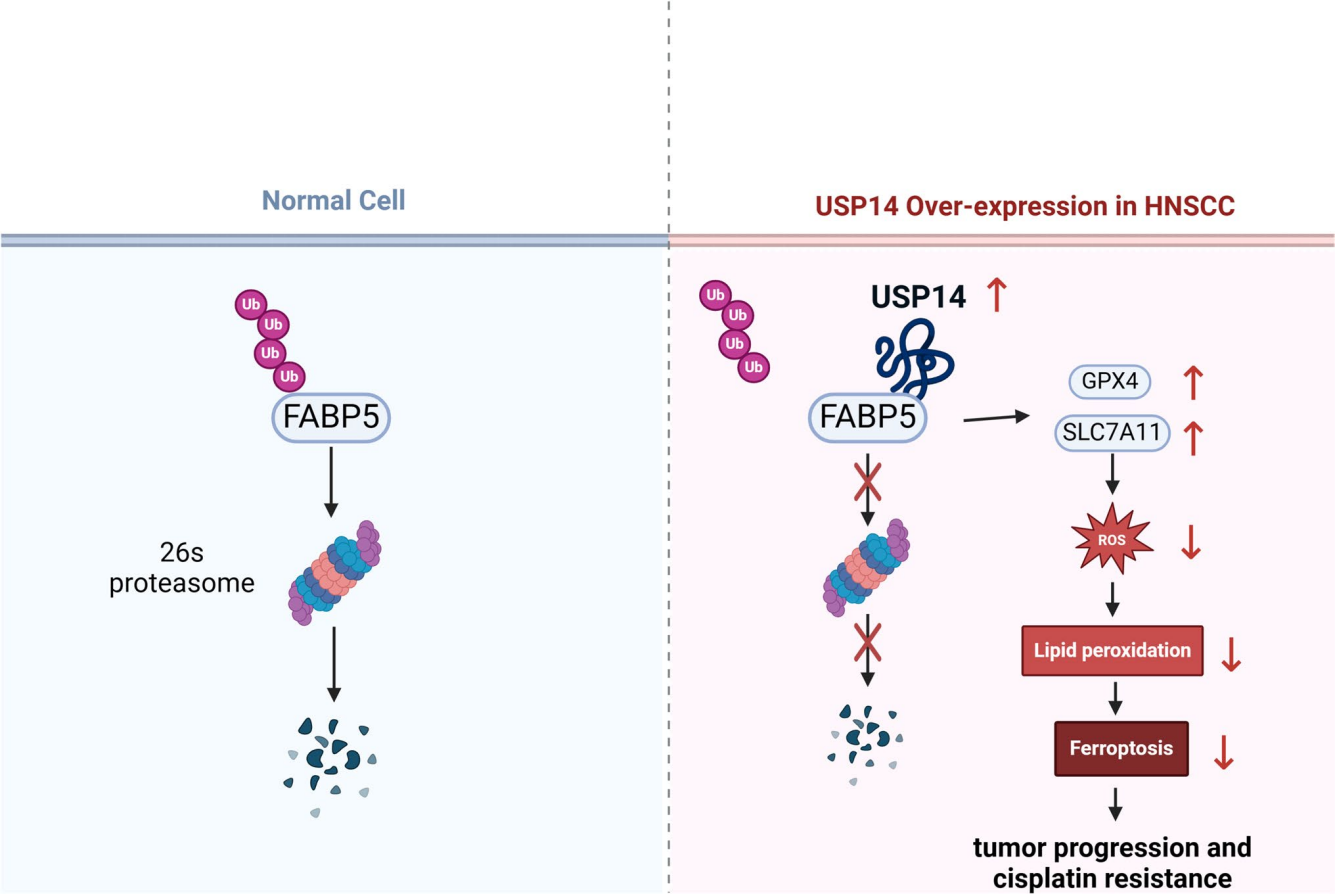
Mechanism diagram

HNSCC epitomizes a highly heterogeneous neoplasm, encompassing varied primary anatomical sites, and has historically lacked efficacious therapeutic targets [1]. This study further indicated that HNSCC tissues have elevated USP14 levels. It was observed that the silencing of USP14 expression notably affected the susceptibility of HNSCC cells to cisplatin, manifesting in considerably decrease in cell viability and increase in ferroptosis. Moreover, the targeted decrease in USP14 expression in HNSCC cells led to a pronounced suppression of their malignant progression both in vitro and in vivo. Similarly, USP14 was also demonstrated to increase the radiotherapy sensitivity of HNSCC, but no attention was paid to drug resistance [30]. Through subsequent mass spectrometry analyses, we found that USP14 can interact with FABP5 protein, facilitating its deubiquitination and consequent stabilization. Further, the overexpression of FABP5 within USP14-depleted cells resulted in a significant reduction in malignant biological behavior among HNSCC cells (Fig. 8).

USP14 is a multifaceted deubiquitinating enzyme, attributable to its extensive repertoire of substrates, and plays pivotal roles in a variety of biological functions. To date, multiple signaling pathways in which USP14 mediates cancer progression and chemotherapy resistance have been identified. For example, USP14 promotes resistance to cisplatin of ovarian cancer by increasing the stability of BCL6 protein [31]. USP14 is related to chemotherapy resistance in patients with metastatic bladder urothelial carcinoma and may potentially be a focus for addressing chemotherapy resistance [32]. Knockdown of USP14 triggers cisplatin induced apoptosis by blocking the Akt and ERK signalling pathways, thereby rendering gastric cancer cells more susceptible to cisplatin [33]. USP14 regulates the progression of many different tumor types, including colorectal cancer, by mediating cancer cell proliferation, apoptosis, and cell cycle arrest [34–37]. In this study, we further explored the potential of USP14 to promote malignant progression and modulate sensitivity towards cisplatin therapy of HNSCC cells. This finding suggested that a vast signalling network centered on USP14 influences numerous facets of oncogenesis. For comprehensive elucidation of the oncogenic functionality of USP14, systematic identification and functional analysis of its substrates are imperative. Herein, FABP5 was identified as a novel substrate of USP14, which contributes to tumor formation by maintaining the stability of oncoproteins. This discovery enriches the existing knowledge of the significant impact of USP14 in the field of cancer research.

To investigate if USP14 facilitates HNSCC progression through the upregulation of FABP5 expression, we overexpressed FABP5 in USP14 knockdown cell lines and found that the malignant phenotype of the cells was restored. Indeed, a substantial body of research supports the oncogenic role of FABP5 in different types of cancer. For example, CCAT1 has been demonstrated to mediate the nuclear translocation of FABP5, subsequently promoting lung adenocarcinoma progression [38]. Furthermore, FABP5 is implicated in the promotion of epithelial-mesenchymal transformation, lymphangiogenesis, and cervical cancer lymph node metastasis through the reprogramming of fatty acid metabolism [39]. Additionally, both in vitro and in vivo studies have revealed the activation of the PPAR signalling pathway by FABP5 in bladder cancer, underscoring its tumorigenic capacity [40]. Studies have shown that in pancreatic neuroendocrine neoplasms, FABP5 activates the PI3K/Akt/mTOR signalling pathway, leading to enhanced lipid metabolism and cellular proliferation [41]. In gastric cancer, FABP5 has been reported to enhance tumor growth, invasion, and migration [42]. Moreover, FABP5 is reportedly associated with ferroptosis. The use of the FABP5 inhibitor SBFI26 leads to cellular accumulation of fatty acids, which triggers excess ferrous ions and subsequent lipid peroxidation, thereby inducing ferroptosis (28). Consistent with previous findings, our study shows that FABP5 is crucial in the progression of HNSCC and is controlled by USP14. The suppression of USP14 decreases the protein levels of GPX4 and SLC7A11 via FABP5, increasing the concentration of ferrous ions and lipid peroxidation in cells, elevating cellular ROS levels, and causing ferroptosis. This renders HNSCC cells more susceptible to cisplatin therapy.

## Conclusions

Our research sheds light on the mechanism by which USP14 contributes to chemotherapy resistance in HNSCC, highlighting its potential as a valuable therapeutic target for treating this disease. There is, however, a requisite for further inquiries to unravel the intricate mechanistic roles of USP14 and FABP5 in the oncogenic evolution of HNSCC and to ascertain their viability as therapeutic targets. In addition, exploring the functional significance of USP14 and FABP5 in various cancer types will enhance our overall comprehension of their functions in cancer biology on a broad scale.

## Supplementary Information

Below is the link to the electronic supplementary material.

Supplementary file1 Supplementary Figure 1: Correlation between USP14 and ferroptosis-related genes. (A) The expression level of 25 ferroptosis-related genes in HNSCC with TCGA database. (B) A correlation analysis between highly expressed ferroptosis-related genes and the USP gene families (TIF 17502 KB)

Supplementary file2 Supplementary Table 1: The representative proteins interacting with USP14 detected by mass spectrometry (DOCX 14 KB)

Supplementary file3 Supplementary Table 2: Correlation between clinical patterns and FABP5 expression in 88 cases of HNSCC (DOCX 15 KB)

## Data Availability

The transcriptome data were obtained from the TCGA database.
