# Supplementary figures and images for: USP14 targets FABP5-mediated ferroptosis to promote proliferation and cisplatin resistance of HNSCC
Source: Clin Transl Oncol. 2025 Feb 10;27(8):3485–500. doi: 10.1007/s12094-025-03857-6 (PMC12259754; doi:10.1007/s12094-025-03857-6)

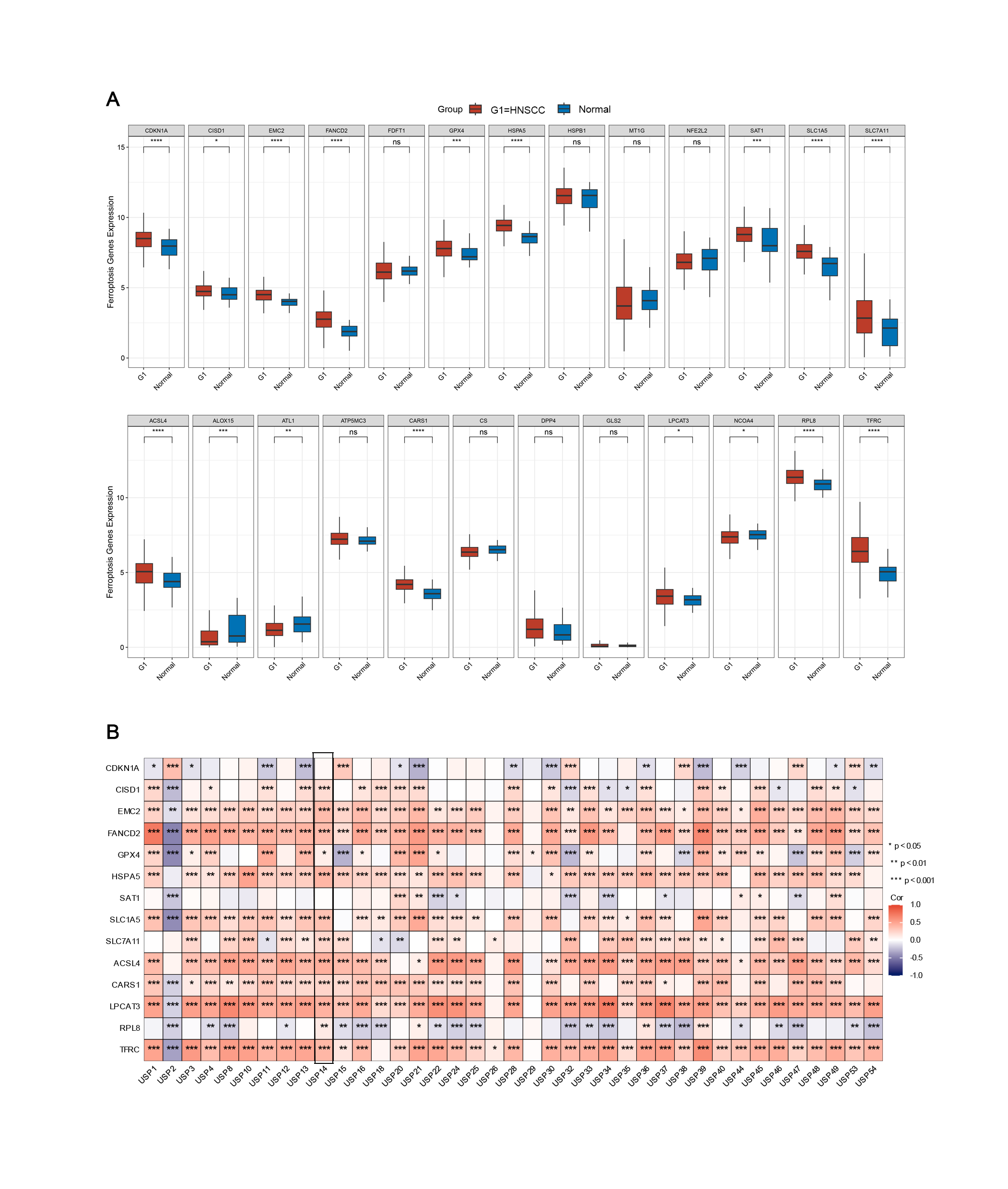

Supplement: Supplementary file 1 — Supplementary file1 Supplementary Figure 1: Correlation between USP14 and ferroptosis-related genes. (A) The expression level of 25 ferroptosis-related genes in HNSCC with TCGA database. (B) A correlation analysis between highly expressed ferroptosis-related genes and the USP gene families (TIF 17502 KB) [file 12094_2025_3857_MOESM1_ESM.tif]
