# Supplementary material for: USP14 targets FABP5-mediated ferroptosis to promote proliferation and cisplatin resistance of HNSCC
Source: Clin Transl Oncol. 2025 Feb 10;27(8):3485–500. doi: 10.1007/s12094-025-03857-6 (PMC12259754; doi:10.1007/s12094-025-03857-6)
Supplement: Supplementary file 2 — Supplementary file2 Supplementary Table 1: The representative proteins interacting with USP14 detected by mass spectrometry (DOCX 14 KB) [file 12094_2025_3857_MOESM2_ESM.docx]

Supplementary table1. The representative proteins interacting with USP14 were detected by mass spectrometry.

| Protein ID | Protein description |
| --- | --- |
| H2AZ1 | Histone H2A.Z |
| RPS26 | 40S ribosomal protein S26 |
| CASP14 | Caspase 14, apoptosis-related cysteine peptidase |
| SSR1 | Signal sequence receptor subunit alpha |
| PKP2 | Plakophilin-2 |
| RAB7A | Ras-related protein Rab-7a |
| SERPINB3 | Serpin B3 |
| FABP5 | Fatty acid-binding protein 5 |
| HNRNPH1 | Heterogeneous nuclear ribonucleoprotein H |
| RBM28 | RNA binding motif protein 28 isoform 1 |
| SPIN | Spindlin, isoform CRA_a |
| DSC1 | Desmocollin-1 |
