# Supplementary material for: USP14 targets FABP5-mediated ferroptosis to promote proliferation and cisplatin resistance of HNSCC
Source: Clin Transl Oncol. 2025 Feb 10;27(8):3485–500. doi: 10.1007/s12094-025-03857-6 (PMC12259754; doi:10.1007/s12094-025-03857-6)
Supplement: Supplementary file 3 — Supplementary file3 Supplementary Table 2: Correlation between clinical patterns and FABP5 expression in 88 cases of HNSCC (DOCX 15 KB) [file 12094_2025_3857_MOESM3_ESM.docx]

Supplementary Table2. Correlation between clinical patterns and FABP5 expression in 88 cases of HNSCC

| Valuable | Number | FABP5 high expression  (n=68) | FABP5 low expression  (n=20) | P value |
| --- | --- | --- | --- | --- |
| Age |  |  |  | 0.541 |
| <60 | 52 | 39 | 13 |  |
| ≥60 | 36 | 29 | 7 |  |
| Gender |  |  |  | 0.199 |
| Male | 85 | 66 | 19 |  |
| Female | 3 | 2 | 1 |  |
| T stage |  |  |  | 0.042* |
| T1-T2 | 44 | 30 | 14 |  |
| T3-T4 | 44 | 38 | 6 |  |
| Clinical stage |  |  |  | 0.190 |
| I-II | 25 | 17 | 8 |  |
| III-IV | 63 | 51 | 12 |  |
| Lymph node metastasis |  |  |  | 0.046* |
| Yes | 48 | 41 | 7 |  |
| No | 40 | 27 | 13 |  |
